# Research on key factors influencing Chinese designers’ use of AIGC: An extension based on TAM and TRI

**DOI:** 10.1371/journal.pone.0314306

**Published:** 2025-02-19

**Authors:** Yu Yao, Xiang Wang, Kaiqiang Sun

**Affiliations:** 1 Design and Craft Graduate School, Hongik University, Seoul, Korea; 2 Institute of Art and Design, Nanjing Institute of Technology, Nanjing, China; 3 Design and Craft Graduate School, Hongik University, Seoul, Korea; Tianjin University, CHINA

## Abstract

With the rapid development of AI intelligent technology, AIGC can bring an innovative revolution to art creation, providing designers with unlimited possibilities but also challenges. These challenges affect the willingness to adopt and constrain the sustainable development of AIGC. The purpose of this study is to analyse the factors of designers’ adoption intention behaviours. This study reconstructed the research model by combining the factors of AIGC technology characteristics and interactivity, technology acceptance model, technology readiness model, etc. The empirical study was conducted from the dual perspectives of AIGC application characteristics and designers’ psychology, in order to predict the factors that predict designers’ behavioural intentions to use AIGC. In this study, a questionnaire survey was conducted among designers in China and 462 valuable responses were received. Through structural equation modelling (SEM) analysis, the study found that: (1) AIGC’s technical features and interactivity positively affect perceived ease of use, and perceived usefulness, but the interactive features do not directly affect perceived usefulness; perceived ease of use and perceived usefulness positively affect designers’ intention to adopt AIGC applications; (2) optimism and innovation positively affect technical features and designers’ intention to adopt; Insecurity negatively affects designers’ willingness to adopt, and insecurity does not affect technical features; discomfort does not affect designers’ technical features and willingness to adopt. This study further extends the theoretical models of TAM(Technology Acceptance Model) and TRI(Technology Readiness Model), provides a theoretical basis for studying designers’ adoption behaviour of AIGC, and enriches the application groups and domains of the theoretical models of TAM and TRI. The results of this study provide inspiration for the development, design, and marketing of AIGC applications, contributing to the realisation and further adoption of AIGC applications, as well as to the professional development of designers.

## 1. Research background

Artificial Intelligence has been defined as "a system that can correctly interpret external data, learn from that data, and use that learning to achieve specific goals and tasks through flexible adaptation [1].With the introduction of AlphaGo in 2015 and ChatGPT in 2022, AI technology has gained a lot of attention after the release of OpenAI’s application called Chat Generation Pre-Training Transformer (ChatGPT) in 2022.AI has created new possibilities and made many opportunities possible in areas including technology (interpretation and information processing), business (decision-making and AI-enabled automation), education (smart tutors and personalised learning), healthcare (smart health and AI diagnostics), and the arts and humanities (human-centred design and cultural proximity) [2].According to Precedence (2023) [3] study, the global market size of generated AI was $10.79 in 2022 and is expected to reach around $118.06 by 2032, with a CAGR of 27.02% from 2023 to 2032.Artificial Intelligence generates content technology, which is trained through big data and generates predictions based on the data to provide users with efficient and personalised service content.

Under the development and promotion of the new era, art design and artificial intelligence have found a new development fit, and a series of new development situations have arisen under the integration of artificial intelligence and art design. Artificial intelligence design is still very different from traditional design methods [4]. Many aspects of design work have been redefined by the application of AIGC technology. Designers provide creative input, feedback and guidance to the AI system, while the AI assists the user to create by providing verbal, visual and decision-making support. Liang et al., (2020) [5] argued that the application of AIGC technology in the design industry focuses on automating the process, generating prototypes quickly and providing customised designs to help designers to quickly iterate on ideas and create a personalised experience based on theuser needs to create a personalised experience. Yin et al., (2023) [6] argued that the Midjourney tool, as a commonly used assistive tool for designers to quickly visualise design models, can effectively improve design efficiency and stimulate creative thinking.

Under the guidance of AIGC, it makes the design field more quality, more efficient, and makes the design content more logical. AIGC has evolved dramatically in the understanding of natural language under the acceleration of new research paradigms as well as algorithmic advances, and there is a substantial performance in handwriting, speech recognition, and language comprehension in these language generating models [7]. For the development of design has also created opportunities, ChatGPT, DALLE-2, Midjourney and other generative artificial intelligence to design creation has brought an innovative revolution, providing designers with unlimited possibilities, through the algorithms to automatically generate images, text, music, video and other content, not only for the designers to provide a new conceptualisation of the tools, but also will reshape the designer’s way of working.

With the rapid development of AIGC technology, designers are faced with unprecedented opportunities and challenges. The emergence of new AIGC technology allows designers to express ideas and realise designs in an unprecedented way, which stimulates their creativity and desire to explore. Kelly (2022) [8] mentions in his article that "they (AI) can createrelevant and understandable new things but, at the same time, completely unexpected". Such unpredictability can cause designers to start experimenting more with AIGC tools, exploring the possibilities offered by the new technology and expecting to create more unique and outstanding works. However, AIGC applications also bring certain anxiety, uneasiness and challenges to designers. On the one hand, in the face of the impact of new technologies, designers may worry that their skills are outdated and unable to adapt to new ways of creating. This anxiety may lead designers to hold some resistance to the new technology or to doubt their own creative ability. On the other hand, the emergence of new technologies also requires that designers designers must have the willingness and ability to learn continuously [9].This also creates learning pressure and a sense of urgency for designers. These factors will constrain the sustainable development of AIGC applications.

Traditional theories of adoption intention models are not strong in explaining the application of AIGC, and related studies on designers’ adoption are not sufficient to reveal the mechanism of designers’ adoption behaviour. It is impossible to explore the influencing factors of AIGC tool use from more perspectives. Liu & She et al. (2024) [10] proposed a multimodal fusion and sales forecasting model, which introduces the interactive and technical features of AIGC into the TAM model and TRI model. These technical features not only enhance product functionality, but also indirectly influence users’ behavioural intentions by improving the interactive experience with users. Based on this line of thinking, in this study, AIGC technical features, interactive features, and designers’ psychological factors are included as key variables in the technology acceptance and usage behaviour model, which further enriches the understanding of the AIGC technology adoption process.

Therefore, this study extends the technology acceptance model and the technology readiness index model to comprehensively analyse the key factors affecting designers’ adoption of AIGC by combining AIGC technology features, interaction features, and other factors. This study not only fills the theoretical gaps in the application of AIGC in the design field, but also provides new ideas and methods for the deep integration and synergistic development of designers and AI, which helps to promote the development of the design field in the direction of intelligence, personalisation and efficiency.

## 2. Literature review

### 2.1 Research status

AIGC is a breakthrough technological development. Tencent Research Institute (2023) [11] believes that AIGC (Artificial Intelligence Generated Content) models such as Stable Diffusion, DALL-E2 and MidJourney have sparked a craze in the field of AI mapping. The emergence of these models marks the deep penetration of AI into the field of art.

Through previous research [12], many scholars have begun to pay attention to the development of AIGC in the field of art. Current research is mainly divided into four areas:

(1) Verifying the advantages of AIGC technology. Most related studies use empirical analysis to measure the effects of AIGC technology on various aspects, such as design efficiency [13], creative expression [14], design interaction [15,16], creative outcomes [17], design strategy and innovation confidence [18]. In particular, there are many studies in specific fields such as fine arts and product design;

(2) Exploring the risks of AIGC technology. The explosive growth of AIGC in a short period of time has led to shortcomings in the standardised use of AIGC and has also concealed many hidden dangers. Elali & Rachid (2023) [19] stated that the use of AIGC tools involved in the design process can create risks of infringement and piracy. Dugan et al. (2023) [20] stated in their article that the use of AIGC tools in the design process provides false and incorrect information, and also stated in their research that artificial intelligence can promote violent information and spread incorrect content. Finally, Sallam (2023) [21] stated that human misuse of AI tools can lead to dependency on the tools and make people lazy;

(3) The application of AIGC technology in art and design. For example, Xu et al. (2023) [22] stated in their research that artificial intelligence has made initial attempts in art, and listed artificial intelligence generation systems such as video generation system Runway, image processing system Toolkit, social media system IO, music system Amper Music, and artistic image system Dall-E2. Cetinic & She (2022) [23] outlined the application of artificial intelligence (AI) in art analysis and creation, emphasising its importance in understanding existing artworks and generating new artworks, and looking ahead to the potential future impact of AI technology in the arts. Mazzone & Elgammal (2019) [24] explore the role of AI in artistic creation, arguing that AI-generated artworks constitute art, and advocate human-machine collaboration to expand creative potential, highlighting the contribution of AI in modelling and analysing artistic styles.

(4) Investigate the current situation of AIGC adoption intentions. Existing research has shown that various factors have a significant impact on people’s willingness to adopt AIGC technology. Gao & Xie (2023) [25] pointed out that perceived usefulness and ease of use are the two main factors influencing the adoption of AIGC technology. Their research shows that when users believe that AIGC technology can significantly improve their work efficiency and is easy to use, they are more likely to adopt the technology. Beaudry & Pinsonneault (2005) [26] also pointed out that the complexity of the technology and the learning curve are important factors influencing the willingness to adopt AIGC. Some users hesitate because they find it difficult to master AIGC technology. Meanwhile, Wang & Chen (2024) [27] explored the influence of perceived attributes and trust on designers’ behavioural intentions from the perspective of perceived risk in their study on the integration of TAMs and TPB. They found that despite the potential benefits of AIGC, designers are reluctant to adopt AIGC due to insufficient trust in the technology, especially in terms of data security and privacy. Referring to the ’gatekeeper network model’ proposed by Wu & Zhai et al. (2023), the study analysed the key influencing factors of AIGC technology adoption among designers by extending the TAM and the Technology Readiness Model. It was found that in the process of AIGC technology adoption, the technology must not only be easy to use and practical, but also effectively connect internal and external resources through the platform and the role of the "gatekeeper" [28].

In conclusion, this study identifies several gaps in the existing research on AIGC adoption intention, as evidenced by an analysis of relevant literature.

(1) The TAM model and adoption intention have not been sufficiently examined in relation to the technical and interactive characteristics of AIGC. As a novel application, AICG exhibits technical characteristics such as autonomous learning capacity, high flexibility and customisability, in addition to interactive characteristics including intelligent interaction, real-time feedback and adjustment, and multimodal interaction. However, a significant number of literature sources have not provided a comprehensive examination of the impact of AIGC characteristics on perceived ease of use, perceived usefulness, and adoption intention.

(2) The extant research concentrates on the technical functions and application effects, whereas there is comparatively little discussion of the psychological state and emotional factors of designers when adopting AIGC technology. The role of psychological factors in moderating the relationship between AIGC technology characteristics and interactive characteristics, as well as usage intentions, remains an unaddressed research gap.

(3) There is a paucity of in-depth analysis of the psychological and emotional factors of designers when using AIGC technology, particularly with regard to the research on creative autonomy, self-efficacy and technological readiness.

(4) Despite the existing literature discussing the impact of perceived usefulness and perceived ease of use on behavioural intention through the TAM model, existing research lacks a detailed analysis of the deeper influencing mechanisms behind behavioural intention. For instance, there is a dearth of systematic empirical analysis examining the direct and indirect impact of AIGC technology’s automation and personalised design capabilities on designers’ attitudes towards its use, and the subsequent translation of these attitudes into actual usage behaviour.

(5) Despite existing literature discussing the application of AIGC technology in design, there is a paucity of analysis concerning the specific effects of the technology’s technical and interactive characteristics on designers’ perceived usefulness and ease of use. The majority of studies do not provide a comprehensive analysis of the impact of these characteristics on designers’ behavioural intentions and creative processes in practice. Furthermore, the role of psychological factors, such as optimism and insecurity, in designers’ acceptance of AIGC technology has not been sufficiently addressed, particularly in the context of integrating the TAM and TRI models.

In light of these shortcomings, the present study will: (1) This study will conduct an in-depth exploration of the technical and interactive characteristics of AIGC, revealing their impact on designers’ perceived ease of use and perceived usefulness. This will contribute to the existing literature on this topic by filling the gaps in knowledge in this area. (2) It incorporates psychological factors, such as optimism and innovativeness, to analyse their impact on designers’ attitudes and behavioural intentions, thereby addressing a gap in research on designers’ psychological and emotional factors. (3) By combining the TAM and TRI models, the study presents a systematic analysis of the relationship between AIGC technical characteristics and designers’ intentions to use, thereby addressing the limitations of existing models.

Consequently, this study will integrate the aforementioned factors, including AIGC technical characteristics, interactive characteristics, perceived ease of use, perceived usefulness, and the designer’s psychology, to comprehensively examine their influence on designers’ adoption of AIGC. While addressing these gaps through empirical analysis, it also offers a novel perspective on understanding designers’ behaviour in using AIGC and provides theoretical support for the future development of AIGC.

### 2.2 TAM model

The TAM model, fully known as the Technology Acceptance Model, was proposed by Davis in 1989.The TAM model, with its valid and robust characteristics, is one of the most commonly used models in current research and is often used to explain users’ acceptance of new technologies and, or how to use a particular technology [29]. In the TAM model, perceived ease of use and perceived usefulness together influence whether or not a user accepts anytechnology in terms of usage imagery and attitudes, and indirectly influence actual usage behaviour [30]. In the TAM model, attitude towards use, as the third component, is an important factor used to judge an individual’s acceptance of a new technology [29]. Gao and Xie (2023) [25] state that designers are more likely to adopt AIGC technology when they perceive that the technology can improve work efficiency and is simple to use. This shows that the attitude towards use directly affects the willingness to actually use the new technology. Perceived ease of use and perceived usefulness are two factors that directly or indirectly influence the adoption attitude of users [31,32]. Where perceived ease of use shows a positive correlation for perceived usefulness, i.e., if a technology is perceived as easy to use, the degree to which it is perceived as useful is also higher [33].

Many scholars have used the TAM model in AI research [34–36], and it has been proven to be effective in predicting and explaining users’ acceptance and adoption behaviours of AI, and AIGC applications are a type of AI, so existing TAM research models and empirical methods can be applied to this study.

### 2.3 AIGC technology characteristics

Whether it is able to improve the efficiency of the user’s work, it has a high value of use for the user of a particular system. Tencent Research Institute (2023) [10] argues that the perceived value of AI-generated content (AIGC) to the designer community is multidimensional, and that the most basic ability of AIGC is to generate content, including text, images, video, code, 3D content, or "multi-modal content" in which several media types are exchanged and combined with each other."Jin & Ryu (2022) [30] believe that the processing of image information can be transmitted with visual information, which increases the persuasive power of imagery communication. It is due to this technical characteristic of AIGC, when designers use ai tools, it can greatly improve the design efficiency of designers. And in the design process to provide better creative combinations, especially interdisciplinary aspects, can make up for the designer in other knowledge areas, transform the designer’s design thinking [14]. It can be seen that the technical characteristics of AIGC focus on its own functionality and task execution capabilities, emphasising the performance of the system in generating content and processing tasks. The main focus is on the functionality and output results of the system itself and emphasises the performance of the system in generating content and processing tasks. Therefore, when designers adopt AIGC, the technical characteristics mainly affect perceived usefulness.

In the context of this study, the technical characteristics of AIGC are (1) AIGC’s high efficiency, which greatly improves the efficiency of designers’ content production; (2) AIGC’s freedom, which allows designers to personalise themselves using AIGC; (3) AIGC’s proactivity, which recognises and understands designers’ needs and intentions, and then automatically generates content that meets users’ requirements; (5) AIGC’srichness, which can continuously improve the quality and diversity of generated content; (5) AIGC automation, which can automatically complete the whole process of design creativity, planning to content generation.

### 2.4 AIGC interaction characteristics

AIGC deeply participates in a variety of creative design activities at the same time, further stimulating the effectiveness of human-computer collaboration. The interactive characteristics of AIGC are mainly reflected in its ability to effectively interact with and feedback from designers or other systems [5]. AIGC is realised through the use of AI techniques and algorithms, enabling AIGC to understand and respond to various inputs and make intelligent adjustments and feedbacks [24].AIGC interaction can better understand designers’ design needs, improve the utility of design and user experience, and at the same time provide designers with more room for innovation [15,16]. In the context of this study, AIGC interaction characteristics refer to the degree of personalised and intelligent interaction experience that the AIGC application satisfies the design needs of the designer through AI algorithms so that the designer can feel the personalised and intelligent interaction experience. It includes: (1) the interaction of AIGC applications can imitate human communication and has "social characteristics"; (2) the interaction of AIGC applications can provide a personalised experience; (3) the interaction of AIGC applications has a fast response speed; and (4) the interaction of AIGC applications has the initiative and intelligence.

The interactive features of AIGC tools significantly improve designers’ work efficiency, creative expression and overall work experience; they enable designers to focus more on creative expression and design innovation, improve work efficiency and creation quality, and give full play to their creative potential. Therefore, the interactive features more affect the designers’ operating experience and perceived ease of use, i.e., the comfort and ease of operation during the use process [37].There is a major impact on perceived ease of use when designers adopt AIGC.

### 2.5 Perceived interaction characteristics vs. technical characteristics

In AIGC research, technical characteristics and perceived interactivity are the two dimensions that are used to analyse the psychology of designers. Technical characteristics focus on the capability and functionality of the technology itself, paying more attention to the aspect of the capability and performance of the technology or tool in performing the task. Perceived interaction characteristics, on the other hand, generally focus on the designer’s subjective experience, i.e., the designer’s experience when interacting with the AIGC system. This characteristic emphasises the system’s ability to understand the designer’s intention quickly and accurately, and to provide feedback and adjustments in a flexible way. It mainly enhances the designer’s comfort in the process of operation and improves the efficiency of creation.

It can be seen that these two dimensions play different roles in the TAM, with the technical characteristics focusing on the functionality and output results of the system itself, and emphasising the system’s performance in generating content and handling tasks. Perceived interaction characteristics, on the other hand, mainly affect the designer’s operating experience and system ease of use, and emphasise the process of interaction between the designer and the system. Therefore, although the improvement of interaction characteristics can be a good operating experience for designers, it does not mean that new technologies and systems also have high technical characteristics. For example, a system may have a high degree of intelligent interaction capabilities, but there are technical limitations in generating high-quality content.T echnical characteristics primarily impact perceived usefulness, whereas perceived interactivity tends to more directly impact a designer’s perceived ease of use; therefore, analysing the two separately allows for a better understanding of the different ways in which the interaction experience and the technical capabilities each impact a designer’s willingness to use.

### 2.6 The impact of technology readiness on designers’ psychology

Technology readiness is a multidimensional psychological structure that portrays two completely opposite psychological tendencies when people are faced with new technologies, i.e., the driver and the inhibitor. The driver encourages people to use the new technology, while the inhibitor discourages people from using it, and both of them work together to determine the individual’s tendency to adopt the new technology [38,39].

Technology readiness contains four dimensions, optimism, innovativeness, discomfort and insecurity. Optimism is the individual’s maintaining an optimistic and positive attitude towards technology, i.e., the user believes that the use of new technology will increase efficiency and make work and life easier. Innovativeness is an individual’s sense of leadership towards technology, where the individual believes that he or she can master a new technology and enjoy the process of its service. Discomfort is the individual’s lack of control over the technology, perceived oppression resulting in mental anxiety and confusion, i.e., the individual perceives the technology as being too complex, the learning time becomes longer, and confidence in mastering the new technology diminishes. Insecurity is the individual’s mistrust of the technology and will have doubts about its ability to work when used. This concern often arises when an individual uses a new technology for the first time, creating doubts about what the unknown brings [38].

In the context of this study, the psychological disposition of different designers is a key factor influencing their adoption of AIGC. Therefore, this study uses technology readiness to delve deeper into the influence of designers’ psychological disposition factors in adopting AIGC. The study shows that designers’ optimism and pursuit of innovation in the face of new technologies make them more willing to embrace AIGC tools, especially in terms of enhancing work efficiency and reducing repetitive labour.

## 3. Research hypotheses and methodology

### 3.1 Research hypotheses

#### 3.1.1 The relationship between interactive features of AIGC and perceived usefulness and ease of use

Previous research has shown that rapid response to questions allows users to make better use of artificial intelligence systems [39]. The most important aspect of AIGC interactivity is human initiative, which can be referred to as machine initiative or AI initiative. AI products can independently perceive the external environment, think, plan and act without human intervention [40]. This means that designers can interact with these AI tools and explore their ideas or concepts in depth with AI tools.

Perceived usefulness refers to the extent to which users subjectively believe that AIGC can improve their work performance or be helpful in their lives. If AIGC can accurately understand the designer’s needs and effectively provide relevant services, designers will perceive its usefulness, thereby increasing their willingness to use it.

At the same time, the AIGC application can autonomously perceive the designer’s information needs, so that the designer can perceive the commands to be implemented through a real-time description of the environment and status [41]. This autonomy allows users to perceive a fast, accurate, efficient and effective response to information without having to manually interact with it, thereby increasing the perceived usability of the AIGC application. This shows that improving interactivity increases the intuitiveness and engagement of the user experience, which in turn increases the utility (perceived usefulness) and usability (perceived ease of use) of the technology. In short, good interaction design not only makes the technology easy to use, but also improves its practical help to users and increases users’ positive acceptance of AIGC [24].

Therefore, the following hypotheses are proposed in this study:

Hypothesis H1a: The interactive features of AIGC have a positive effect on perceived usefulness.
Hypothesis H1b: The interactive features of AIGC have a positive effect on perceived usefulness.

#### 3.1.2 The relationship between technical features of AIGC and perceived usefulness and ease of use

As an application based on artificial intelligence technology, AIGC can generate various content such as text, images and voice by training a large amount of data to learn the characteristics and patterns of human language. This feature of high efficiency, speed and scalability gives AIGC significant advantages in improving work efficiency and releasing productivity [16].

In the application of AIGC, because AIGC can generate content with natural language and creative design and imitate human creative styles, it can bring high value to designers in many scenarios. AIGC can use the generated content to improve work efficiency and optimise the creative process, which makes designers perceive the application of AIGC as useful. Previous studies [2,17,42] have shown that the technical features of AIGC affect the perceived usefulness of users. AIGC technology is also constantly being optimised and improved to provide a more concise and intuitive interface and process, reducing the learning cost and difficulty of use for users. The technical features of AIGC help to improve the perceived usefulness of AIGC.

Therefore, this study proposes the following hypotheses:

Hypothesis H2a: AIGC technical features will positively affect the perceived usefulness of AIGC applications.
Hypothesis H2b: AIGC technical features will positively affect the perceived usefulness of AIGC applications.

#### 3.1.3 The relationship between perceived usefulness and perceived ease of use and attitude and intention to use

Perceived usefulness refers to the extent to which a particular system is believed to improve the user’s work efficiency or enable the user to perform better at work [43]. In the field of design, it is expressed as the extent to which designers can reduce the time spent on design and produce high quality design results through the use of specific technologies [44]. Perceived ease of use refers to the extent to which a new technology is easy and convenient for users to operate without having to spend a lot of time learning. Numerous studies have shown that users’ perceived usefulness plays a key role in determining whether they accept and adopt a technology or system. In particular, for AI image generators, users are more likely to adopt and use the technology if they perceive it as a valuable tool that provides instant information, personalised recommendations and efficient drawing functions [25]. Perceived ease of use and perceived usefulness are two important concepts in the TAM model, and both factors directly or indirectly influence users’ attitude towards adoption [31,32]. In particular, the concept of perceived ease of use emphasises the comfort and accessibility of using the technology. Research also shows that users’ perceptions of the ease of use of a technology or system significantly influence their acceptance and adoption intentions. If the learning or operating process of a new technology is easy, they will perceive the AI image generator as a practical and valuable tool because they can easily use its various functions during use, thus increasing their perception of the software’s usefulness [45]. Users will then be more likely to accept and use it. Therefore, perceived ease of use directly affects perceived usefulness [43].

Therefore, this study proposes the following hypotheses:

H3a: Perceived usefulness of the AIGC application will positively affect attitude towards its use.
H3b: Perceived usefulness of the AIGC application will positively affect intention to use.
H4a: Perceived ease of use of the AIGC application positively affects attitude towards its use.
H4b: Perceived ease of use of the AIGC application will positively affect perceived usefulness.

#### 3.1.4 The relationship between attitude towards use and intention to use

The TAM model suggests that the adoption of new technologies is determined by the user’s usage attitude, which is the critical factor in the user’s adoption of new technologies [46,47]. Usage attitude towards a new technology results from the emotions experienced by the user during the service process, and these emotions can strongly influence the willingness to use the technology [48]. Therefore, user acceptance of information systems is directly determined by the subjective willingness to act, which is jointly determined by the user’s attitude and the perceived usefulness of the information system. Empirical evidence shows that attitude is positively correlated with intention to act [2,49,50].

Therefore, this study proposes the following hypothesis:

Hypothesis 5: The intelligence of AIGC application characteristics will positively affect the behavioural intention of AIGC application characteristics.

#### 3.1.5 Relationship between technology readiness and AIGC technology characteristics and behavioural intention

Technology readiness as a psychological state includes two drivers: optimism and innovativeness, and two inhibitors: discomfort and uncertainty [51]. The drivers can increase users’ willingness to adopt new technologies, while the inhibitors limit adoption attitudes. The final usage intention is the result of the combined effect of both [52]. If the bias towards conservatism in technology adoption is too strong and people are reluctant to become innovators or take risks, this will affect their willingness to use. At the same time, perceptions of the complexity, risk and uncertainty of the technology can also strongly influence the likelihood of its use [53]. In this study, designers apply different psychological states to AIGC, which will directly affect the technical characteristics and adoption intentions of AIGC. In general, optimistic users are more likely to adopt new technologies because they enjoy the excitement of exploring the technology and the sense of control it brings. Existing research shows that technology acceptance (TR) influences people’s intention to use technology products or services. Among these, positive factors such as optimism and innovativeness tend to have a positive effect on intention to use, while negative factors such as discomfort and uncertainty tend to have a negative effect on new technologies and intention to use [54].

Therefore, the following hypotheses are proposed in this study:

H6a: Optimism positively affects the technical characteristics of AIGC applications.
H6b: Optimism will positively affect the behavioural intention of AIGC applications.
H7a: Innovativeness positively influences the technical characteristics of AIGC applications.
H7b: Innovativeness positively influences the behavioural intention of AIGC application
H8a: Uncomfortableness negatively influences technical characteristics of AIGC use
H8b: Uncomfortableness negatively influences behavioural intention to use AIGC
H9a: Uncomfort negatively influences the technical characteristics of AIGC application
H9b: Uncertainty negatively influences behavioural intention to use AIGC

### 3.2 Research structure

**Fig 1:**
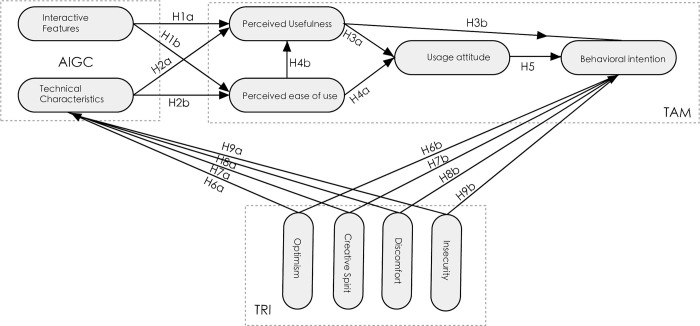
Research model.

This research builds on previous studies by integrating the TAM and the TRI with factors such as AIGC technological characteristics and interaction characteristics. In light of the aforementioned hypotheses, this study puts forth the research model depicted in Fig 1.

### 3.3 Definition and measurement of variables

The principal variables in this research model encompass Interactive features, AIGC Technological Characteristics, Perceived Usefulness (PU), Perceived Ease of Use (PEOU), Attitude Toward Use (AT), Behavioral Intention (BI), Optimism (Op), Innovativeness (CS), Discomfort (Di), and Insecurity (In). The measurement indicators for these variables are presented in Table 1 for reference.

**Table 1:** Operational definitions of variables and reference scales.

variant	Operational definitions	coding	Measurement items	Literature sources
Interactive features	The AIGC app meets designers’ design needs through artificial intelligence algorithms, thus allowing designers to feel the degree of personalised and intelligent interactive experience.	IF1	AIGC application interactions mimic human communication and have "social characteristics"	(Tsikriktsis N,2004) [55],(Qu Z,2023) [56]
		IF2	AIGC application interactions can provide a personalised experience	
		IF3	AIGC application interactions have fast response times	
		IF4	AIGC application has the interactive initiative, intelligent	
Technical characteristics	Technical characteristics exhibited by AIGC in handling complex design tasks during designers’ use of AIGC applications.	TC1	AIGC technology enables rapid content generation and greatly improves the efficiency of content production.	(Zhang C,2023) [57],(Jin Z,2023) [58]
		TC2	AIGC technology offers a high degree of freedom in content generation and can be personalised according to the user’s needs.	
		TC3	AIGC recognises and understands the needs and intentions of the user and then automatically generates content that meets the user’s requirements.	
		TC4	AIGC technology is able to continuously improve the quality and diversity of generated content.	
		TC5	AIGC has a highly automated production capability that automates the entire process from ideation and planning to content generation.	
Perceived usefulness	Perceived usefulness refers to the subjective evaluation of its value and utility as perceived by the designer when using the AIGC application.	PU1	The use of AIGC technology can improve work and learning efficiency and reduce the time it takes to complete tasks	(Davis F D,1989) [43],(Venkatesh V,2000) [59],(Chatterjee S,2021) [60]
		PU2	Using AIGC technology can help me to get innovative ideas in my study and work.	
		PU3	Use of AIGC technology is useful for performing study and work tasks	
Perceived ease of use	Perceived ease of use refers to the designer’s perception of the ease of use of an AIGC application when using it specifically.	PEOU1	Learning and using AIGC technology was easy for me!	(Davis F D,1989) [43],(Venkatesh V, 2000) [59],(Yousafzai S Y,2007) [61]
		PEOU2	The AIGC technique was easy for me to become proficient in.	
		PEOU3	AIGC’s interaction between me and AIGC technology is clear and concise	
		PEOU4	AIGC technology works for me.	
Attitude	Positive or negative psychological tendencies held by designers when considering whether or not to adopt new technologies for AIGC applications.	AT1	I’m excited about the new AIGC technology	(Davis F D,1989) [44],(Na S,2022) [62]
		AT2	I’ve had a lot of fun using the new technology!	
		AT3	I think new technology is necessary in life or work	
Behavioral intention	Designers’ willingness and inclination to adopt new technologies for AIGC applications.	BI1	I believe the use of AIGC technology is beneficial	(Venkatesh V,2000) [59].(Castiblanco Jimenez I A,2021) [33],(Taylor S,1995) [63]
		BI2	I believe that designing with AIGC technology is worthwhile	
		BI3	I believe that AIGC technology will help me to improve my abilities	
		BI4	I intend to design using AIGC technology	
Optimism	Positive perceptions and attitudes of designers towards AIGC’s application of new technologies.	Op1	I believe that the use of new technology can improve the efficiency of work	(Parasuraman A,2000) [38],(Parasuraman A,2015) [52]
		Op2	When it comes to new technologies, I tend to focus on the positive impacts rather than the potential problems	
		Op3	I believe that the new technology will bring more benefits to the society	
		Op4	I’m happy to try new technologies, even if I don’t fully understand them	
Creativity	The creativity and exploratory spirit of the designers when confronted with the new AIGC technology.	CS1	I enjoy exploring and experimenting with new technologies	(Parasuraman A,2000) [38],(Parasuraman A,2015) [52]
		CS2	In the team, I’m often the first to try new technology	
		CS3	I’m always looking for new technologies to solve problems at work or in life	
Discomfort	Physical and mental discomfort or distress experienced by designers when using the new AIGC technology.	Di1	I feel anxious or uncertain when confronted with new technology	(Parasuraman A,2000) [38],(Parasuraman A,2015) [52]
		Di2	I think it takes a lot of time to learn new technologies	
		Di3	I find it hard to keep up with the fast pace of technology	
		Di4	I worry about making mistakes when using new technology	
Insecurity	Designers using AIGC to apply new technologies develop doubts about what the unknown brings.	In1	I would worry about the reliability of new technology before I started using it	(Parasuraman A,2000) [38],(Parasuraman A,2015) [52]
		In2	I’m concerned about the potential invasion of privacy with the new technology.	
		In3	I feel distrustful of new technologies that are too complex to understand	
		In4	I think the risks of new technologies may outweigh the benefits they bring	

## 4. Data and methods

This study uses a questionnaire and structural equation modelling (SEM) for empirical research. Structural equation modelling is an advanced statistical method that combines the advantages of factor analysis and path analysis. It can simultaneously handle multiple dependent variables, latent variables and their interrelationships, as well as measurement error in these relationships [64]. Just as et al. (2024) used structural equation modelling (SEM) to analyse the predictive value of AIGC in e-commerce [10], SEM can be used in the context of this study to effectively analyse the factors influencing designers’ willingness to adopt AIGC.

### 4.1 Questionnaire design

This study used a questionnaire survey method for empirical research. Before the formal survey, a pre-test was conducted to verify the reliability and validity of the questionnaire. Between 5 January and 17 January 2024, we distributed 60 questionnaires and received 52 valid questionnaires. To improve the reliability of the questionnaire options, we analysed the reliability of the pre-test questionnaire and removed the questionnaire options that did not meet the requirements.

The formal questionnaire was distributed to designers using the AIGC app between January and February 2024. Respondents could access the questionnaire by clicking on the URL link in the questionnaire. In addition to basic personal information, respondents indicated their level of agreement or disagreement on a 7-point Likert scale ranging from 1 (strongly disagree) to 7 (strongly agree). During the survey, designers agreed to complete the questionnaire on a fully informed and voluntary basis, answered the research questions and had the option to withdraw at any time. A total of 513 samples were collected for this study. After removing invalid samples (e.g. logical errors and duplicates), 462 samples remained, giving a validity rate of 90.06%.

In the maximum likelihood method, the ratio of the estimated parameter to the sample size (p:n) should ideally be 1:10 [65,66]. This study has 38 test variables and 462 valid samples, with a sample size of 1:12.16, which exceeds the theoretically recommended value. Therefore, we used SPSS 23.0 and AMOS 17.0 software to analyse the collected valid data.

### 4.2 Scale reliability analysis

Reliability analysis, also known as consistency or stability testing, is a method of examining the stability, consistency, and reliability of measurement results. To guarantee the precision of the measurements, a reliability analysis of the valid data from the questionnaire was performed prior to the commencement of further analysis. In the field of social science research, the Cronbach’s α coefficient is a commonly employed measure for the assessment of reliability. In general, a reliability coefficient above 0.9 indicates excellent reliability, between 0.8 and 0.9 indicates very good reliability, between 0.7 and 0.8 indicates good reliability, between 0.6 and 0.7 indicates acceptable reliability, and below 0.6 suggests the need for revision.

In this study, the Cronbach’s α coefficient was employed to test the reliability of the scales using the SPSS 23.0 statistical software. As illustrated in Table 2, all reliability coefficient values exceed 0.7, thereby indicating that the data’s reliability is of an exceedingly high standard. With regard to the α coefficient if item deleted, no significant increase in reliability was observed upon the removal of any item, thereby indicating that no items should be removed. Furthermore, the corrected item-total correlation (CITC) values for all items exceeded 0.4, indicating strong correlations between the items and acceptable reliability levels. In conclusion, the data exhibits high reliability, as indicated by all reliability coefficient values exceeding 0.7, and is thus suitable for further analysis.

**Table 2:** Cronbach’s alpha reliability analysis.

Dimension	Name	Corrected Item-Total Correlation (CITC)	Alpha if Item Deleted	Dimension’s Cronbach’s Alpha	Overall Cronbach’s Alpha 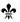
IF1	IF1	0.763	0.848	0.886	0.897
	IF2	0.755	0.851		
	IF3	0.748	0.854		
	IF4	0.734	0.859		
TC1	TC1	0.775	0.879	0.904	
	TC2	0.751	0.884		
	TC3	0.769	0.881		
	TC4	0.792	0.876		
	TC5	0.71	0.893		
PU1	PU1	0.725	0.765	0.843	
	PU2	0.689	0.799		
	PU3	0.71	0.779		
PEOU1	PEOU1	0.743	0.836	0.875	
	PEOU2	0.745	0.835		
	PEOU3	0.713	0.848		
	PEOU4	0.726	0.842		
AT1	AT1	0.722	0.797	0.853	
	AT2	0.719	0.801		
	AT3	0.733	0.786		
BI1	BI1	0.766	0.848	0.886	
	BI2	0.747	0.855		
	BI3	0.723	0.864		
	BI4	0.766	0.847		
Op1	Op1	0.751	0.85	0.884	
	Op2	0.752	0.849		
	Op3	0.758	0.847		
	Op4	0.728	0.858		
CS1	CS1	0.68	0.801	0.84	
	CS2	0.715	0.766		
	CS3	0.716	0.766		
Di1	Di1	0.726	0.86	0.884	
	Di2	0.765	0.845		
	Di3	0.772	0.842		
	Di4	0.729	0.858		
In1	In1	0.764	0.874	0.899	
	In2	0.755	0.877		
	In3	0.784	0.866		
	In4	0.797	0.862		

Standardization Cronbach α coefficient: 0.897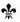.

### 4.3 Descriptive statistics

The demographic characteristics of the sample are presented in Table 3. In terms of age distribution, the majority of the sample falls within the 26–35 years old category, representing 39.61% of the total. With regard to gender, the sample indicates a slight predominance of male respondents, representing 51.30% of the total, while 48.70% of the respondents are female. In terms of educational attainment, over 40% of the sample has obtained a Bachelor’s degree. A total of 20.13% of respondents identified as fashion designers.

**Table 3:** Demographic characteristics.

Category	Option	Frequency	Percentage(%) 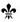
Age	25 and under	70	15.15
	26–35 years	183	39.61
	36–45 years	124	26.84
	46–55 years	61	13.2
	56–65 years	24	5.19
Gender	Male	237	51.3
	Female	225	48.7
Education	Diploma or below	115	24.89
	Bachelor’s degree	193	41.77
	Master’s degree	69	14.94
	PhD	54	11.69
	Postdoc	31	6.71
Occupation	Product Designer	72	15.58
	Graphic Designer	70	15.15
	Environmental Designer	75	16.23
	Architectural Designer	90	19.48
	Fashion Designer	93	20.13
	Jewelry Designer	62	13.42
Monthly Income	Below 5000	46	9.96
	5000–7000	109	23.59
	7000–9000	117	25.32
	9000–11000	124	26.84
	Above 11000	66	14.29
Work Experience	One year or less	63	13.64
	1–5 years	167	36.15
	5–10 years	147	31.82
	10–15 years	61	13.2
	15 years and above	24	5.19
Total		462	100

In terms of monthly income, over 20% of the sample falls within the 9,000–10,000 RMB income bracket. In terms of work experience, the most common period is that which spans one to five years, representing 36.15% of the sample. This is followed by a period of five to ten years, which represents 31.82% of the total.

The sample collected for this study is relatively evenly distributed across various demographic variables, thereby reflecting a balanced representation of the study’s target population.

### 4.4 Validity analysis

#### 4.4.1 KMO and Bartlett’s test

The construct validity of this study was analyzed using factor analysis in SPSS, which verifies whether the measurement scale meets the requirements for the research objectives. The Kaiser-Meyer-Olkin (KMO) test and Bartlett’s test of sphericity were conducted on the relevant variables using the Statistical Package for the Social Sciences (SPSS) to evaluate the suitability of factor analysis.

Validity can be defined as the extent to which a test or measurement tool accurately captures the psychological and behavioural characteristics it is designed to measure, thereby ensuring the accuracy and reliability of the results. In general, a smaller significance level for Bartlett’s test of sphericity (P < 0.05) is indicative of a higher likelihood of meaningful relationships between the original variables. The KMO value is used to assess the suitability of factor analysis by comparing the magnitude of the simple and partial correlation coefficients among items, with a value of 0 indicating no suitability and a value of 1 indicating optimal suitability. The standards for suitability for factor analysis are as follows: A KMO value greater than 0.9 indicates excellent suitability for factor analysis, while a value between 0.7 and 0.9 indicates good suitability, and a value between 0.6 and 0.7 indicates moderate suitability. A value between 0.5 and 0.6 indicates marginal suitability, while a value below 0.5 suggests that factor analysis should not be performed. Bartlett’s test of sphericity is employed to ascertain whether the correlation matrix is significantly disparate from the identity matrix. A significance value of less than 0.05 indicates that factor analysis is an appropriate methodology for the data.

As illustrated in Table 4, the KMO value for this study is 0.913, which exceeds 0.8, indicating that the data are highly suitable for factor extraction (and indirectly suggesting good validity).

**Table 4:** KMO and Bartlett’s test results.

KMO Value		0.859
Bartlett’s Test of Sphericity	Chi-squareapproximation 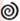 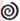	10616.21
	df	703
	p-value	0

#### 4.4.2 Exploratory factor analysis

As can be seen in Table 5, the SPSS factor analysis shows that there are 10 factors with eigenvalues greater than 1 in the questionnaire items. The first factor explains 22.499% of the variance, which is less than the critical standard of 40%. The results show that there is no serious common method bias in the data collection of the questionnaire used in this study [67].

**Table 5:** Explained variance table.

Factor Number	Eigenvalues 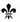			Variance Explained Before Rotation			Variance Explained After Rotation		
	Eigenvalue	Variance Explained (%) 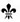	Cumulative (%) 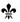	Eigenvalue	Variance Explained (%) 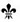	Cumulative (%) 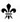	Eigenvalue	Variance Explained (%)	Cumulative (%)
1	8.55	22.499	22.499	8.55	22.499	22.499	3.712	9.768	9.768
2	4.464	11.747	34.246	4.464	11.747	34.246	3.105	8.17	17.938
3	2.732	7.191	41.437	2.732	7.191	41.437	3.021	7.95	25.888
4	2.551	6.714	48.151	2.551	6.714	48.151	3.021	7.95	33.839
5	2.14	5.632	53.783	2.14	5.632	53.783	3.015	7.933	41.772
6	2.048	5.389	59.172	2.048	5.389	59.172	2.982	7.846	49.618
7	1.779	4.681	63.853	1.779	4.681	63.853	2.95	7.762	57.38
8	1.601	4.213	68.066	1.601	4.213	68.066	2.293	6.035	63.416
9	1.447	3.808	71.874	1.447	3.808	71.874	2.282	6.004	69.42
10	1.287	3.388	75.262	1.287	3.388	75.262	2.22	5.842	75.262
11	0.631	1.661	76.923	-	-	-	-	-	-
12	0.581	1.528	78.451	-	-	-	-	-	-
13	0.508	1.337	79.788	-	-	-	-	-	-
14	0.487	1.283	81.071	-	-	-	-	-	-
15	0.473	1.245	82.316	-	-	-	-	-	-
16	0.453	1.191	83.508	-	-	-	-	-	-
17	0.436	1.147	84.654	-	-	-	-	-	-
18	0.42	1.105	85.76	-	-	-	-	-	-
19	0.395	1.04	86.799	-	-	-	-	-	-
20	0.382	1.005	87.804	-	-	-	-	-	-
21	0.364	0.958	88.762	-	-	-	-	-	-
22	0.351	0.924	89.686	-	-	-	-	-	-
23	0.335	0.881	90.567	-	-	-	-	-	-
24	0.32	0.841	91.409	-	-	-	-	-	-
25	0.315	0.828	92.237	-	-	-	-	-	-
26	0.291	0.766	93.002	-	-	-	-	-	-
27	0.286	0.752	93.754	-	-	-	-	-	-
28	0.269	0.708	94.462	-	-	-	-	-	-
29	0.255	0.672	95.134	-	-	-	-	-	-
30	0.254	0.67	95.804	-	-	-	-	-	-
31	0.239	0.628	96.432	-	-	-	-	-	-
32	0.219	0.576	97.008	-	-	-	-	-	-
33	0.202	0.533	97.541	-	-	-	-	-	-
34	0.199	0.523	98.064	-	-	-	-	-	-
35	0.192	0.505	98.569	-	-	-	-	-	-
36	0.19	0.501	99.07	-	-	-	-	-	-
37	0.183	0.482	99.552	-	-	-	-	-	-
38	0.17	0.448	100	-	-	-	-	-	-

Meanwhile, the cumulative variance explained is 75.262%, which indicates that the extracted common factors have high validity. As can be seen from Table 5, a total of 10 common factors were extracted, which is consistent with the previously divided dimensions, further indicating good validity.

In order to better explain the naming of each principal factor, an orthogonal rotation of the factor load matrix was performed using the maximum variance method. The size of the factor load value was used as a criterion for retention and deletion when selecting measurement items. A factor load matrix was generated and factor loads less than 0.4 were deleted. Topics in the final factor arrangement that were in the same column were placed in the same category.

As shown in Table 6, the correlation coefficients of all the research items are higher than 0.4, which means that there is a strong correlation between the research items and the factors, and the factors can effectively extract the information. After ensuring that the factors can extract most of the information of the research items, the correspondence between the factors and the research items is analysed (the absolute value of the factor load coefficient is greater than 0.4, which indicates that the item and the factor have a corresponding relationship).

**Table 6:** Rotated factor loadings.

name	Factor Loadings Coefficients 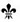									
	Factor 1	Factor 2	Factor 3	Factor 4	Factor 5	Factor 6	Factor 7	Factor 8	Factor 9	Factor 10
IF1	0.083	0.057	0.008	0.057	0.849	0.112	0.123	0.062	0.012	0.035
IF2	0.095	0.094	0.001	0.085	0.837	0.022	0.149	0.031	0.011	0.076
IF3	0.064	0.069	0.05	0.042	0.841	0.057	0.083	0.08	0.042	0.11
IF4	0.121	0.062	0.082	0.1	0.809	0.079	0.121	0.066	0.001	0.106
TC1	0.848	0.03	0.023	0.04	0.112	0.077	0.04	0.087	0.068	0.054
TC2	0.822	0.006	0.067	0.126	0.071	0.122	0.064	0.084	-0.007	0.012
TC3	0.819	0.039	0.029	0.129	0.073	0.087	0.094	0.073	0.06	0.132
TC4	0.835	0.043	0.013	0.068	0.076	0.116	0.117	0.081	0.067	0.129
TC5	0.773	-0.063	0.07	0.123	0.055	0.11	0.065	0.082	0.071	0.101
PU1	0.075	-0.009	0.117	0.094	0.03	0.152	0.065	0.039	0.845	0.105
PU2	0.094	0.006	0.168	0.132	0.007	0.086	0.045	0.148	0.808	0.094
PU3	0.062	0.05	0.037	0.085	0.022	0.107	0.128	0.051	0.841	0.145
PEOU1	0.072	-0.009	0.03	0.085	0.161	0.181	0.808	0.112	0.089	0.045
PEOU2	0.101	-0.044	0.037	0.015	0.114	0.108	0.82	0.119	0.089	0.122
PEOU3	0.126	0.003	0.124	0.126	0.106	0.105	0.798	0.063	0.034	0.098
PEOU4	0.065	0.028	-0.008	0.108	0.119	0.008	0.825	0.164	0.046	0.045
AT1	0.132	0.046	0.015	0.081	0.061	0.06	0.156	0.836	0.083	0.109
AT2	0.086	0.095	-0.015	0.102	0.099	0.11	0.122	0.837	0.073	0.07
AT3	0.177	0.074	0.007	0.067	0.081	0.129	0.175	0.807	0.085	0.153
BI1	0.143	-0.101	-0.02	0.16	0.083	0.824	0.116	0.058	0.041	0.103
BI2	0.113	-0.056	-0.012	0.158	0.05	0.817	0.107	0.085	0.113	0.099
BI3	0.164	-0.113	-0.025	0.123	0.074	0.783	0.081	0.084	0.114	0.086
BI4	0.108	-0.169	-0.012	0.183	0.091	0.791	0.107	0.096	0.122	0.13
Op1	0.098	-0.015	-0.042	0.821	0.07	0.166	0.089	-0.009	0.101	0.138
Op2	0.129	-0.062	0.004	0.824	0.078	0.117	0.059	0.094	0.082	0.119
Op3	0.095	-0.022	0.044	0.829	0.089	0.148	0.096	0.069	0.043	0.138
Op4	0.154	-0.034	0.007	0.798	0.059	0.16	0.091	0.11	0.106	0.019
CS1	0.105	-0.009	0.015	0.127	0.118	0.147	0.125	0.112	0.112	0.796
CS2	0.17	-0.002	0.099	0.148	0.117	0.158	0.114	0.079	0.134	0.798
CS3	0.146	-0.057	0.066	0.146	0.112	0.098	0.073	0.152	0.131	0.804
Di1	0.021	0.149	0.822	0.01	0.031	0.008	0.099	-0.019	0.048	0.105
Di2	0.065	0.137	0.858	-0.038	0.044	-0.039	-0.024	0.038	0.093	0.024
Di3	0.006	0.191	0.85	0.036	0.037	0.004	0.046	0.01	0.054	0.069
Di4	0.093	0.108	0.832	0.007	0.025	-0.033	0.046	-0.017	0.112	-0.034
In1	0.02	0.849	0.161	-0.068	0.068	-0.056	-0.003	0.034	-0.01	-0.02
In2	0.01	0.835	0.126	0.032	0.12	-0.126	-0.007	0.06	0.042	-0.009
In3	0.005	0.854	0.119	-0.11	0.062	-0.128	0.031	0.095	-0.003	0.016
In4	0.015	0.868	0.192	0.015	0.033	-0.079	-0.039	0.019	0.019	-0.047

Rotation Method: Varimax.

### 4.5 Measurement model

#### 4.5.1 Convergent validity

We used AMOS 17.0 to analyse the structural equation model. As a large number of studies use AMOS for analysis, AMOS has proven to be a reliable software for structural equation modelling. Anderson & Gerbing (1988) [68] suggest that data analysis can be divided into two stages. The first stage is the measurement model, which uses maximum likelihood estimation to estimate parameters such as factor loadings, reliability, convergent validity and discriminant validity. The second stage is a convergent validity study based on Hair et al. (1998) [69], Raykov& Marcoulides (2011) [70], Fornell & Larcker (1981) [71] and Chin (1998) [72].

As shown in Table 7. The itemised standardised factor loadings for all variables are greater than 0.7, indicating that each observed variable contributes significantly to the explanation of its latent variable. It should be noted that the CR is greater than 0.8, which is significantly higher than the standard of 0.7. This indicates that the observed variables under each dimension can explain the dimension well. The AVE values in this study are all above the standard value of 0.6, indicating that the convergent validity of the scale is good.

**Table 7:** Summary of determinants analysis.

			STD.Estimate	Estimate	S.E.	C.R.	P	CR	AVE
IF4	<--	interactive features	0.797	1				0.886	0.66
IF3	<--	interactive features	0.806	1.034	0.056	18.384	***		
IF2	<--	interactive features	0.82	1.019	0.054	18.769	***		
IF1	<--	interactive features	0.826	1.013	0.054	18.928	***		
TC5	<--	technical characteristic	0.756	1				0.904	0.654
TC4	<--	technical characteristic	0.846	1.116	0.06	18.664	***		
TC3	<--	technical characteristic	0.82	1.062	0.059	18.039	***		
TC2	<--	technical characteristic	0.796	1.042	0.06	17.443	***		
TC1	<--	technical characteristic	0.823	1.117	0.062	18.109	***		
PU1	<--	perceived usefulness	0.823	1				0.843	0.642
PU2	<--	perceived usefulness	0.781	0.952	0.057	16.744	***		
PU3	<--	perceived usefulness	0.799	0.974	0.057	17.038	***		
PEOU1	<--	perceived ease of use	0.818	1				0.876	0.638
PEOU2	<--	perceived ease of use	0.816	1.06	0.056	18.98	***		
PEOU3	<--	perceived ease of use	0.775	1.012	0.057	17.862	***		
PEOU4	<--	perceived ease of use	0.783	0.99	0.055	18.078	***		
AT3	<--	attitude	0.842	1				0.853	0.66
AT2	<--	attitude	0.793	0.89	0.05	17.843	***		
AT1	<--	attitude	0.801	0.947	0.053	17.985	***		
BI4	<--	behavioral intention	0.838	1				0.886	0.66
BI3	<--	behavioral intention	0.779	0.921	0.049	18.847	***		
BI2	<--	behavioral intention	0.805	0.923	0.047	19.674	***		
BI1	<--	behavioral intention	0.827	0.965	0.047	20.419	***		
Op1	<--	optimism	0.817	1				0.884	0.656
Op2	<--	optimism	0.813	1.052	0.055	19.169	***		
Op3	<--	optimism	0.824	1.069	0.055	19.479	***		
Op4	<--	optimism	0.786	1.039	0.056	18.384	***		
CS1	<--	creativity	0.76	1				0.84	0.637
CS2	<--	creativity	0.824	1.083	0.065	16.548	***		
CS3	<--	creativity	0.81	1.038	0.063	16.383	***		
Di1	<--	discomfort	0.784	1				0.885	0.657
Di2	<--	discomfort	0.829	1.036	0.056	18.632	***		
Di3	<--	discomfort	0.842	1.101	0.058	18.935	***		
Di4	<--	discomfort	0.786	1.03	0.059	17.548	***		
In1	<--	insecurity	0.818	1				0.899	0.691
In2	<--	insecurity	0.807	1.002	0.052	19.45	***		
In3	<--	insecurity	0.842	1.054	0.051	20.575	***		
In4	<--	insecurity	0.857	1.072	0.051	21.052	***		

Overall, all of the measurement items show significance at the 0.001 level (P<0.001), and the standardised load factor values are all greater than 0.6, indicating that overall there is good correspondence between the factors and the measurement items, and the convergent validity is good. The average variance extraction AVE value is >0.5 and the composite reliability CR value is >0.7, indicating good convergent validity.

#### 4.5.2. Discriminant validity

The assessment of discriminant validity was conducted in accordance with the approach proposed by Fornell & Larcker (1981) [71]. The discriminant validity of a construct is established when the square root of the average variance extracted (AVE) for that construct is greater than the absolute value of the correlations between that construct and the other constructs in the model. This suggests that the constructs in the model are distinct from one another.

The results demonstrate that the diagonal values (representing the square roots of the AVEs) exceed the off-diagonal correlation values, indicating that all constructs in this study possess satisfactory discriminant validity (see Table 8). It can therefore be concluded that each dimension in this study exhibits high discriminant validity.

**Table 8:** Discriminant validity of measurement model.

	Interactive features	behavioral intention	technical characteristic	optimism	perceived usefulness	creativity	Perceived ease of use	discomfort	attitude	insecurity
Interactive features	0.812									
behavioral intention	0.240***	0.813								
Technical characteristic	0.269***	0.357***	0.809							
Optimism	0.245***	0.462***	0.333***	0.81						
Perceived usefulness	0.123*	0.345***	0.241***	0.312***	0.801					
Creativity	0.328***	0.422***	0.383***	0.420***	0.409***	0.798				
Perceived ease of use	0.366***	0.347***	0.287***	0.294***	0.268***	0.350***	0.799			
Discomfort	0.122*	-0.027	0.126*	0.033	0.254***	0.164**	0.127*	0.811		
Attitude	0.264***	0.319***	0.350***	0.282***	0.290***	0.401***	0.418***	0.065	0.812	
Insecurity	0.169**	-0.253***	0.036	-0.090†	0.04	-0.035	-0.002	0.381***	0.140**	0.831

The value of AVE square root > ranks maximum indicates good discriminant validity.

### 4.6 Structural model analysis

#### 4.6.1 Model fit indices

A model with a good fit indicates that the model closely reflects the sample. In accordance with the recommendations of Jackson et al. (2009) [66], Kline (2015) [73], Whittaker (2011) [74], Hu & Bentler (1999) [75], and other scholars, this study employed a range of fit indices to assess the fit of the structural model. The selected fit indices are as follows: CMIN, degrees of freedom (DF), CMIN/DF, Normed Fit Index (NFI), Tucker-Lewis Index (TLI), Comparative Fit Index (CFI), Goodness of Fit Index (GFI), Relative Fit Index (RFI), and Incremental Fit Index (IFI).As illustrated in Table 9, the majority of the model fit indices align with the optimal standards, substantiating the model’s commendable fit.

**Table 9:** Model fit indices.

Model Fit	Criteria	Model Fit of Research Model	Judgment
CMIN	-	1210.346	-
DF	-	637	-
CMIN/DF	1~5	1.900	Ideal
RMSEA	<0.08	0.044	Ideal
NFI	>0.90	0.889	Good
TLI	>0.90	0.938	Ideal
CFI	>0.90	0.944	Ideal
RFI	>0.90	0.878	Good
IFI	>0.90	0.944	Ideal

#### 4.6.2 Path analysis

As evidenced in Table 10, when the interactive and technical features influence perceived ease of use, the standardized path coefficients are all greater than 0, and p is less than 0.01. This indicates that the interactive and technical features have a significant positive impact on perceived ease of use.

**Table 10:** Path coefficients.

			STD.Estimate	Estimate	S.E.	C.R.	P	significance
technical characteristic	<--	optimism	0.223	0.205	0.051	4.036	***	statistically significant
technical characteristic	<--	creativity	0.302	0.299	0.058	5.136	***	statistically significant
technical characteristic	<--	discomfort	0.058	0.055	0.051	1.079	0.281	insignificant
technical characteristic	<--	insecurity	0.042	0.038	0.048	0.802	0.423	insignificant
perceived ease of use	<--	interactive features	0.324	0.318	0.051	6.209	***	statistically significant
perceived ease of use	<--	technical characteristic	0.226	0.237	0.053	4.47	***	statistically significant
perceived usefulness	<--	interactive features	0.018	0.018	0.058	0.314	0.754	insignificant
perceived usefulness	<--	technical characteristic	0.202	0.222	0.06	3.697	***	statistically significant
perceived usefulness	<--	Perceived ease of use	0.203	0.212	0.062	3.412	***	statistically significant
attitude	<--	perceived usefulness	0.193	0.2	0.055	3.623	***	statistically significant
attitude	<--	Perceived ease of use	0.379	0.411	0.059	6.985	***	statistically significant
behavioral intention	<--	perceived usefulness	0.173	0.179	0.051	3.484	***	statistically significant
behavioral intention	<--	optimism	0.283	0.295	0.055	5.371	***	statistically significant
behavioral intention	<--	creativity	0.197	0.221	0.061	3.628	***	statistically significant
behavioral intention	<--	discomfort	-0.026	-0.027	0.054	-0.505	0.613	insignificant
behavioral intention	<--	insecurity	-0.241	-0.25	0.052	-4.773	***	statistically significant
behavioral intention	<--	attitude	0.167	0.167	0.049	3.408	***	statistically significant

Note: ***p***<0.05 ***p***<0.01 ***p***<0.001.

The effect of the interactive features on perceived usefulness is not significant (p > 0.05), indicating that there is no impact of the interactive features on perceived usefulness.

When technology features and perceived ease of use affect perceived usefulness, the standardized path coefficients are all greater than 0 and p is less than 0.01, indicating that technology features and perceived ease of use have a significant positive impact on perceived usefulness. When perceived usefulness and perceived ease of use affect usage attitude, the standardized path coefficients are all greater than 0 and p is less than 0.01, indicating that perceived usefulness and perceived ease of use have a significant positive impact on usage attitude. When perceived usefulness, optimism, innovative spirit and attitude towards use affect behavioural intention, the standardized path coefficients are all greater than 0 and p is less than 0.01, indicating that perceived usefulness, optimism, innovative spirit and attitude towards use have a significant positive impact on behavioural intention. In the case of a negative correlation between behavioural intention and insecurity, the standardised path coefficient is less than 0 and p is less than 0.01, indicating that insecurity exerts a significant negative influence on behavioural intention. The effect of discomfort on behavioural intention is not significant at the 0.05 level, indicating that discomfort has no impact on behavioural intention. When optimism and innovative spirit affect the technical characteristics, the standardized path coefficients are all greater than 0 and p is less than 0.01, indicating that optimism and innovative spirit have a significant positive impact on the technical characteristics. In the case of a significant correlation between insecurity and discomfort and the technical characteristics, the p-value is not significant when greater than 0.05. This indicates that there is no impact of insecurity and discomfort on the technical characteristics.

### 4.7 Hypothesis interpretation

**Fig 2:**
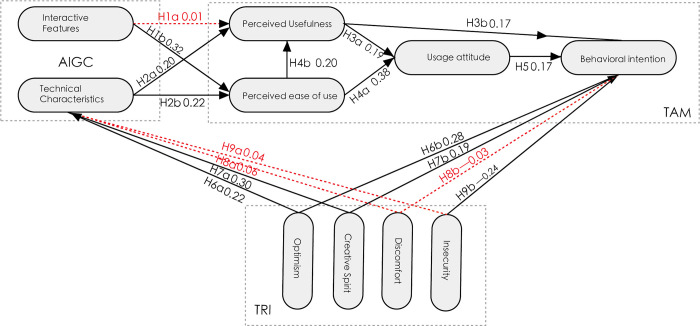
Hypothetical path coefficients diagram.

As illustrated in Fig 2, the regression coefficients of the structural equation model (SEM) employed in this study are presented. A higher coefficient indicates that the independent variable exerts a more pronounced influence on the dependent variable.

The hypotheses H1b, H2a, H2b, H3a, H3b, H4a, H4b, H5, H6a, H6b, H7a, and H7b are supported by the model, indicating that the relationships between variables were found to be significant.

The hypotheses H1a, H8a, H8b, and H9a are not supported, indicating that these proposed relationships did not have a significant effect on the model.

## 5. Discussion

This study extends the technology acceptance model and the technology readiness model, and combines factors such as AIGC technology characteristics and interaction characteristics to explore designers’ willingness to adopt AIGC applications.

First, H1b and H2a and H2b are valid, which shows that the interaction characteristics have an effect on perceived ease of use, and that the technical characteristics of AIGC have an effect on both perceived usefulness and perceived ease of use. It can be seen that the interaction characteristics and the technical characteristics of AIGC have an important effect on perceived ease of use and perceived usefulness [76]. In this study, the interactive features refer to the technology that enables the AIGC to understand and empathise with human language. This technology exhibits human-like psychological activity characteristics and social characteristics by simulating the way humans communicate with each other [77]. Fast response, AI initiative, and autonomous perception and action capabilities without human intervention are considered key factors in improving user satisfaction and enhancing the interactivity of artificial intelligence systems [78]. This not only satisfies the user’s need for information. It also allows designers to quickly become proficient and reduce learning time without being familiar with AIGC tools, thereby improving learning efficiency and user experience.

The technical characteristics of AIGC are useful for designers to perceive that AIGC tools can improve design efficiency and design quality. At the same time, the technical features also reduce the entry barrier of AIGC tools and reduce the time designers spend learning AIGC tools. This is consistent with the findings of Gao & Xie (2023) [25] and others. It is also consistent with the findings of Wu & Wang et al. (2023) [79] in the e-commerce domain. Our research also shows that AIGC technology not only improves design efficiency, but also enhances designers’ creativity through personalised and customised services. Future research can focus more on how AIGC technology can be adapted for use in design practices in different cultural and market environments.

This study found that H1a was null, indicating that the interactive features of AIGC do not affect perceived usefulness, i.e. the interactive features have no effect on designers’ perceptions that AIGC can improve design efficiency or enhance innovation. This contradicts the findings of Gill et al. (2024) [39]. This study believes that under the premise that the two main features of AIGC tools, namely interactivity and technical features, have an impact, designers can quickly experience the technical experience brought about by AIGC. However, in the process of improving design efficiency with AIGC tools, designers can only experience it through the technical features of AIGC, and the interactive features will not directly affect the perceived usefulness of AIGC.

The establishment of H3a, H3b, H4a and H4b indicates that both perceived usefulness and perceived ease of use have a significant positive impact on designers’ usage attitudes and behavioural intentions. After perceiving the practical usefulness and ease of use of AIGC, designers are more likely to form positive usage attitudes, which ultimately influence their usage intentions. This finding is consistent with the core assumptions of the TAM model [28,43], that is, users’ perceptions of the usefulness and ease of use of a technology are a key factor in determining their intention to use it. Establishing H5 indicates that attitude towards use has a significant positive effect on behavioural intention. The more positive designers’ attitudes towards AIGC technology, the more likely they are to use the technology in their actual work. This finding further validates the efficacy of the TAM model and supports the core idea of attitude determining behaviour in technology acceptance theory.

The fact that H6a, H6b, H7a and H7b are all valid indicates that optimism and innovativeness have a significant positive impact on both AIGC technology characteristics and behavioural intention, which is consistent with previous research [37,38,52]. Designers with an optimistic and innovative spirit are more likely to accept and use AIGC technology. They are more likely to explore new technologies and believe that these technologies can bring positive change.

H8a and H8b did not hold, indicating that discomfort had no significant effect on AIGC technology characteristics and behavioural intentions, which is inconsistent with previous research [53,54]. This result shows that although designers may feel uncomfortable when faced with new technologies, this emotion is not enough to significantly affect their perceptions of technology characteristics or their intention to use them. Designers may have become accustomed to constantly changing design tools and technologies, so that discomfort has not become a major barrier.

H9b states that uncertainty has a significant negative effect on behavioural intention, but H9a does not, meaning that it has no significant effect on technology attributes. This shows that although uncertainty does not directly affect designers’ perceptions of AIGC technology characteristics, it does significantly reduce their willingness to use it. Designers are more likely to reduce their reliance on AIGC technology when they are concerned about data security and privacy issues. Therefore, reducing designers’ uncertainty is crucial to improving the adoption rate of AIGC technology.

Previous research has extensively explored the perceived benefits of AIGC technology to users [77]. This has led to the conclusion that technology acceptance is generally predicted by perceived expectations of new technology. However, there is a lack of research and analysis on the psychological characteristics of designers, which makes it impossible to predict from the perspective of designers’ own characteristics what kind of technology can adapt to their characteristics.

Therefore, this study examines the acceptance of new technology not only from the technical perspective of AIGC, but also from the psychological characteristics of designers. To some extent, the path coefficients and the relationship between designers and new technologies in the newly proposed model have been updated to take into account the types and characteristics of designers. The results of this study are not only used to update the coefficients and relationships of the theoretical model of AIGC, but also to analyse the relationship between designer characteristics and technology acceptance. On the other hand, this study has constructed a valuable structural model and provided possible directions, thus laying the foundation for further analysis.

This study is a good empirical study, but given the limitations of this study, the following are some possible directions for future research.

First, this study mainly focuses on the key factors influencing the adoption of AIGC by Chinese designers and may not fully represent the diversity of the global designer community. Differences in cultural and economic backgrounds may affect the adoption of AIGC technology. Due to the vast geographical area of China, the designer community in this study is mainly concentrated in some developed provinces and cities such as Shanghai and Jiangsu, and designers in other regions were not studied. Future research can conduct more in-depth studies on designer groups of different nationalities and regions.

Second, this study did not further subdivide designers of different professions. Designers from different professions have certain differences in their attitudes towards AIGC. Future research could look more closely at designers from different professions.

At the same time, there are significant differences in the technology of different AIGC applications and different application areas (such as video generation and 3D generation). Different types of AIGC application technology may influence designers’ willingness to adopt them. In the future, further research can be conducted on designers’ willingness to adopt different AIGC applications.

Finally, this study examines the adoption of new AIGC technologies from the technical aspects of AIGC and the characteristics of designers. The adoption of AIGC applications by designers may be influenced by a variety of other factors, such as the social environment, economics, emotions and ethics. These factors can be further explored in future research.

## 6. Conclusion

(1) Theoretical contribution

This study not only investigated the effectiveness of AIGC technology in practical applications, but also obtained real-life feedback from designers on their use of AIGC technology through a questionnaire survey. These data provide a scientific basis and practical guidance for the promotion and application of AIGC technology in the field of design. It provides a reference for design education and training and helps designers better respond to the challenges of technological change. It provides a scientific basis and practical guidance for the promotion and application of AIGC technology in the field of design.

Theoretically, this study extends the application of the TAM to the designer community. By integrating TAM and TRI theories, a more comprehensive theoretical framework is constructed to explain Chinese designers’ acceptance of AIGC technology. This framework not only considers the characteristics of the technology itself, but also incorporates personal psychological traits such as optimism, innovation and uncertainty into the model, and shows how these factors affect designers’ acceptance and willingness to use AIGC technology. This innovative perspective enriches the content of technology acceptance theory and provides new directions for future research.

At the same time, our research identifies key influencing factors. The results show that: (1) The technical features and interactivity of AIGC have a positive impact on perceived ease of use and perceived usefulness. Improving the usability and practicality of AIGC technology significantly improves designers’ willingness to use it. This finding highlights the key role of user experience in the technology adoption process and suggests that developers and technology providers should focus on optimising interaction design and functional usability. (2) The role of optimism and innovation in promoting technology adoption suggests that companies or educational institutions should focus on cultivating and motivating designers’ innovative spirit and reducing their fear of using technology when promoting AIGC technology. (3) The negative impact of uncertainty on adoption needs to be addressed. Ensuring data security and user privacy will be key measures to address designers’ concerns and improve the effectiveness of technology use. Technology providers can increase designers’ confidence in AIGC tools and promote their widespread use by improving system security measures and providing users with clear data usage policies.

In conclusion, this study has theoretically extended the scope of the technology acceptance model and identified the unique influencing factors in the designer community. Our research findings have important academic value for promoting the application of AIGC technology and enhancing designers’ innovation capabilities, and also provide theoretical support for the digital transformation of the design industry.

(2) Practical contribution

This study not only investigates the effectiveness of AIGC technology in practical applications, but also obtains real-life feedback from designers on their use of AIGC technology through questionnaires. This data will provide a scientific basis and practical guidance for the promotion and application of AIGC technology in the field of design. With the continuous development of AIGC technology, the design industry is undergoing a transformation from traditional design tools to intelligent design tools. The study provides theoretical support and practical guidance for this transformation, promoting innovation and upgrading of design tools. It provides a reference for design education and training, helping designers to better respond to the challenges posed by technological change. It provides a scientific basis and practical guidance for the promotion and application of AIGC technology in the field of design. Practical cases, such as the successful promotion of Adobe Firefly, show that simplifying the learning curve and personalising interactions can reduce designers’ resistance and accelerate technology adoption. MidJourney and Stable Diffusion have reduced users’ sense of insecurity through transparent privacy policies and training resources, which has improved tool adoption [80]. At the same time, this study provides a reference for education and training, suggesting the development of targeted training programmes based on the psychological readiness of designers, such as the inclusion of hands-on sessions for tools like Runway AI in the curriculum, to help designers adapt more quickly to new technologies. In addition, the study provides suggestions for policymakers to promote joint government-enterprise support for designers’ technological transformation and innovative development, and provides guidance for the development of future design tools, suggesting the addition of real-time feedback and natural language processing functions to improve user experience and work efficiency.
